# A multifactorial study on nutritional status, binge eating and physical activity as main factors directly influencing body weight in Parkinson’s disease

**DOI:** 10.1038/s41531-017-0018-0

**Published:** 2017-05-22

**Authors:** Andrea Bril, Santiago Perez-Lloret, Malco Rossi, Sofía Fariña, Pierre Morisset, Laura Sorrentino, Micaela Iglesias, Alex Medina Escobar, Patricio Millar Vernetti, Daniel Cerquetti, Marcelo Merello

**Affiliations:** 1 Movement Disorders Section, Neuroscience Department, Raul Carrea Institute for Neurological Research (FLENI), Buenos Aires, Argentina; 2 Institute of Cardiology Research, University of Buenos Aires, CONICET-ININCA, Buenos Aires, Argentina; 3 Argentine National Scientific and Technological Research Council (CONICET), Buenos Aires, Argentina

## Abstract

Weight lossisa multifactorial disorder commonly affecting Parkinson’s disease patients. The aim of this study was to investigate the relationship between body weight, nutritional status, physical activity, and Parkinson’s disease-related factors. A total of 114 consecutive Parkinson’s disease patients without dietary restrictions were evaluated prospectively with respect to: nutritional status (Mini Nutritional Assessment), physical activity level (Yale Physical Activity Survey), MDS-UPDRS score, olfactory function, depression, cognitive functionand impulse-control disorders, among other variables. Structural equation modeling was used to build multivariate models and to calculate standardized regression weights (srw) for pairs of variables, which are homologous to correlation coefficients, taking into account the effects of all other variables in the model. Sixty (53%) patients were males. Mean age was 66.1 ± 9.8 years and mean disease duration was 8.3 ± 5.6 years. Longer disease duration was negatively related to nutritional status (srw = −0.25; *p* = 0.01). UPDRS II + III score was associated with reduced cognitive function (srw = −0.39; *p* = 0.01), which was positivelyrelated to nutritional status (srw = 0.23; *p* = 0.01). Finally, nutritional status was positively related to body weight (srw = 0.22, *p* < 0.01). Binge eating and physical activity were also directly and positively related to body weight (srw = 0.32; *p* = 0.001 and srw = 0.23; *p* = 0.001). Nutritional status, binge eating and physical activity were directly and independently related to body weight in our sample of Parkinson’s disease patients. Therefore, physicians should actively explore nutritional status and binge eating in Parkinson’s disease patients to avoid alterations in body weight regulation. Effects of physical activity should be further explored.

## Introduction

Many Parkinson’s disease (PD) patients experience weight changes, and up to 52%^1^ of patients will lose weight during the course of disease.^2–6^ Previous studies have attempted to identify primary mechanisms involved in this multifactorial process, including motor and non-motor symptoms influencing energy balance, ultimately affecting body weight. Long disease duration,^7^ disease severity,^8^ and female gender^9^ appear to be established body weight determinants. In addition, olfactory dysfunction,^10^ dysphagia, impaired hand–mouth coordination,^11^ decreased cognitive function,^9, 12^ altered neuroendocrine regulation of appetite,^13^ reduced leptine levels,^5^ intestinal dysmotility, and Levodopa side effects,^14, 15^ have also been related to inadequate energy intake; whereas raised metabolic rate at rest^7^ has been linked to enhanced muscle activity due to tremor, rigidity or dyskinesia which may contribute to increase energy expenditure. Other pharmacological factors might include dopamine agonist intake and impulse-control disorders, such as binge eating.^16^ Weight gain has also been observed after deep brain stimulation.^17^

In general, previous studies have focused on specific factors leading to weight loss. This study aimed to prospectively investigate the relationship between body weight, nutritional status, physical activity, and PD-related factors, in a relatively large sample of PD patients applying structural equation modeling (SEM).

## Results

A total of 114 PD patients were examined. Four (3.5%) patients were underweight, 35 (30.7%) had normal body weight, 45 (39.5%) were overweight, and 30 (26.3%) were obese. According to Mini Nutritional Assessment (MNA), 74 (64.9%) patients were adequately nourished, 32 (28.1%) were at risk of malnutrition, and eight (7.0%) were malnourished. Table 1 summarizes subject demographics, medications, and clinical features.

**Table 1:** Demographic and clinical features of PD patients

Total numbers	114
Males (%)	60 (53%)
Age (*y*)	66.1 ± 9.8
Body weight (kg)	77.3 ± 17.9
Body mass index	29.4 ± 4.6
Mini Nutritional Assessment	24.7 ± 3.8
Yale Physical Activity Survey (kcal/day)	657.3 ± 569.2
Diabetes (%)	15 (13%)
Hypothyroidism (%)	18 (16%)
PD disease duration (*y*)	8.3 ± 5.6
MDS-UPDRS-I	7.8 ± 5.2
MDS-UPDRS-II	10.0 ± 7.3
MDS-UPDRS-III	21.2 ± 11.2
MDS-UPDRS-IV	5.4 ± 3.9
H&Y stage: median (range)	2 (1–4)
Motor complications (%)	46 (40%)
Motor fluctuations (%)	41 (36%)
Dyskinesias (%)	29 (25%)
Levodopa use (%)	84 (74%)
Dopamine agonist use (%)	87 (76%)
LEDD (mg)	689.5 ± 506.4
Constipation (%)	58 (51%)
Dysphagia (%)	24 (21%)
Hyposmia (%)	81 (71%)
Beck Depression Inventory	11.2 ± 7.2
Impulse control disorders (%)	25 (22%)
Binge eating (%)	8 (7%)
Montreal Cognitive Assessment	26.0 ± 2.7
PDSS-2	14.6 ± 12.1
Antidepressants	10.53%
Drugs for dementia	2.63%

*MDS-UPDRS* movement disorders society-unified Parkinson’s Disease rating scale, *LEDD* Levodopa equivalent daily dose, *PDSS-2* Parkinson’s disease sleep scale 2

Pearson’s coefficient calculations showed significant correlation between body weight and Montreal Cognitive Assessment(MOCA) score (*r* = 0.21, *p* < 0.05), nutritional status (*r* = 0.39, *p* < 0.01), physical activity (*r* = 0.27, *p* < 0.01), Beck Depression Index (*r* = −0.20, *p* < 0.05), and binge eating (*r* = 0.32, *p* < 0.01). Correlations with other variables were not significant.

**Fig. 1:**
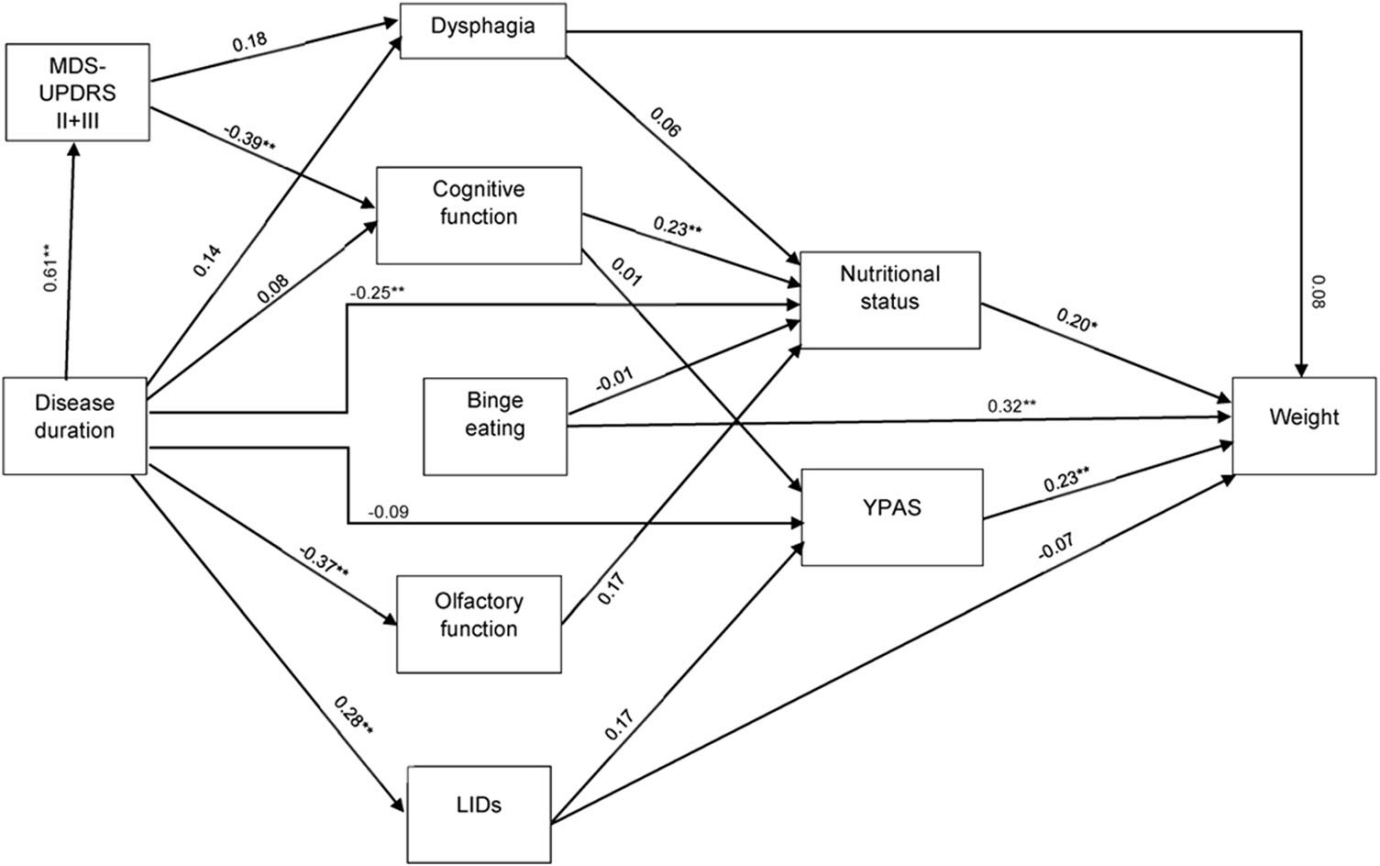
Flow chart connecting PD-related factors to body weight, as obtained using structural equation modelling. Age-adjusted, gender-adjusted, and height-adjusted standardized regression weights are presented for relevant pairs of variables. *p* < 0.05, **p < 0.01

SEM found several statistically significant direct and indirect relationships between clinical variables and body weight or nutritional status. Indicators of model validity were as follows: *χ* ^2^ = 459 *p* < 0.001, NFI = 0.54, CFI = 0.70, RMSEA = 0.08. Figure 1 illustrates the main links between clinical variables. Longer disease duration showed negative correlation to nutritional status (srw = −0.25; *p* = 0.01), which in turn had positive correlation to body weight (srw = 0.19; *p* = 0.01). PD severity was associated with reduced cognitive function (srw = −0.39; *p* = 0.01), which had in turn positive correlation to nutritional status (srw = 0.23; *p* = 0.01). Binge eating was directly and positively linked to body weight (srw = 0.32; *p* = 0.001). There was no influence of hyposmia, depression or swallowing impairment on body weight or nutritional status. A complete list of age-adjusted, height-adjusted, and gender-adjusted standardized regression weights for all pairs of variables tested can be found on Table 2.

**Table 2:** Standardized regression weight calculated with structural equation modeling

Independent variable	Dependent variable		Standardized regression weight		*P*-value
PD duration	Beck Depression Index		−0.184		0.131
PD duration	Binge eating		0.068		0.554
PD duration	Constipation		0.109		0.380
**PD duration**	**Dopamine agonists daily equivalent dose (mg)**		**0.395**		**0.001**
PD duration	Dysphagia		0.140		0.248
**PD duration**	**Levodopa daily dose (mg/kg)**		**0.228**		**0.025**
PD duration	Levodopa-induced dyskinesias		0.282		0.048
**PD duration**	**MNA**		−**0.252**		**0.006**
PD duration	MOCA		0.077		0.531
**PD duration**	**Olfactory function**		−**0.371**		**<0.001**
PD duration	PDSS-2		−0.071		0.561
PD duration	Body weight		0.042		0.719
**PD duration**	**UPDRS II** **+** **III**		**0.614**		<0.001
PD duration	YPAS		−0.093		0.393
**UPDRS II** **+** **III**	**Beck Depression Index**		**0.535**		<0.001
UPDRS II + III	Constipation		0.151		0.269
**UPDRS II** **+** **III**	**Dopamine agonists daily equivalent dose (mg)**		−**0.290**		**0.032**
UPDRS II + III	Dysphagia		0.184		0.096
**UPDRS II** **+** **III**	**Levodopa daily dose (mg/kg)**		**0.529**		**<0.001**
**UPDRS II** **+** **III**	**MOCA**		−**0.388**		**0.005**
Dopamine agonists daily equivalent dose (mg)	Binge eating		0.093		0.334
Levodopa daily dose (mg/kg)	Binge eating		−0.049		0.662
Dopamine agonists daily equivalent dose (mg)	Levodopa-induced dyskinesias		0.180		0.198
Levodopa daily dose (mg/kg)	Levodopa-induced dyskinesias		0.061		0.708
Binge eating	MNA		−0.014		0.862
Dysphagia	MNA		0.06		0.475
**MOCA**	**MNA**		**0.234**		**0.004**
Olfactory function	MNA		0.175		0.082
MOCA	PDSS-2		−0.015		0.875
**UPDRS II** **+** **III**	**PDSS-2**		**0.426**		**0.004**
Levodopa-induced dyskinesias	YPAS		0.170		0.209
MOCA	YPAS		0.009		0.921
Antidepressants	Body weight		−0.055		0.419
Beck Depression Index	Body weight		0.122		0.129
**Binge eating**	**Body weight**		**0.320**		**<0.001**
Constipation	Body weight		0.029		0.687
Diabetes Mellitus	Body weight		−0.026		0.703
Dopamine agonists daily equivalent dose (mg)	Body weight		−0.036		0.637
Hypothyroidism	Body weight		−0.027		0.695
Levodopa daily dose (mg/kg)	Body weight		−0.158		0.124
Levodopa-induced dyskinesias	Body weight		−0.073		0.550
**MNA**	**Body weight**		**0.200**		**0.016**
MOCA	Body weight		0.105		0.171
Olfactory function	Body weight		0.055		0.519
PDSS-2	Body weight		0.125		0.102
UPDRS II + III	Body weight		−0.050		0.758
**YPAS**	**Body weight**		**0.230**		**0.002**
Dysphagia	Body weight		0.082		0.540

Standardized regression weight adjusted for age, gender, and height

Significant associations highlighted in bold type

*YPAS* Yale physical activity survey, *MoCA* Montreal cognitive assessment

## Discussion

This study is to the best of our knowledge, one of the largest prospective and comprehensive studies on factors affecting body weight in PD patients, and appears to confirm a multi-step, multifactorial pathophysiological mechanism underlying body weight disturbances in PD. We found nutritional status, binge eating, and physical activity to be the only variables independently and directly associated with body weight in the multivariate model. PD duration, UPDRS II + III score and cognitive impairment also influenced body weight, but through indirect influence on nutritional status.

In this study, a powerful statistical technique (SEM) was employed to evaluate association between body weight and a large number of PD-related and non-related factors. Detailed description of the technique can be found elsewhere^18^. One of the main advantages of this approach is that variables can simultaneously serve as dependent and independent factors, therefore, permitting modelling of multi-step pathways and networks.

Our study has been limited by lack of stage V Hoehn & Yahr participants, making it impossible to establish whether determinants proposed by previous studies, such as swallowing impairment or severe motor symptoms increasing energy expenditure, had any impact on body weight during later, wheelchair-bound PD stages. In addition, SEM model “fit” indexes were in general lower than expected, indicating presence of other body weight determinants, not included in this study.The regression weights revealed in general mild to moderate associations, which might mean that there are other factors affecting weight that were not accounted for in this study.

Body weight fluctuations in PD patients have been attributed to a variety of causes,^13^ one being nutritional disorders. According to MNA results, 35.1% of patients in this study were either malnourished or at risk of malnutrition. These results are in line with another study^19^ which detected risk of malnutrition in 35% of PD patients measured by the same questionnaire. We found nutritional status was one of the few factors directly related to body weight, and mediated by effects of disease duration and severity, also confirming previous findings.^8, 9^ Despite the apparent importance of nutritional status, it is not normally assessed in clinical practice. Contrary to findings in this study, use of levodopa has been related to worse nutritional status.^14^ We did observe levodopa dose was related to PD duration and severity, which were in turn related to altered nutritional status. Further studies are needed to explore if levodopa has any direct effect on body weight regulation.

We also found nutritional status was affected by cognitive function, confirming previous findings.^8, 12^ Nutritional status assessment has not been analyzed in many studies on body weight, limiting interpretation of results. In this study, UPDRS II + III had negative influence on cognitive status, as has been observed before.^8^ Interestingly, in our model, direct link between UPDRS II + III score and body weight was not significant, suggesting impact of PD severity on weight is mediated by cognitive status. Dementia or visual hallucinations may produce weight loss in advanced PD due to reduced appetite or neuroendocrine dysfunction that causes negative energy balance.^19^

Binge eating was found to be directly and positively associated with body weight in the multivariate SEM analysis. Previous studies showed that dopaminergic agents, particularly dopamine agonists, are a common cause of impulse-control disorders such as binge eating.^16, 20^ However, we found no relationship between dopamine agonists and binge eating, using SEM or classical statistical methods, possibly because physicians sometimes reduce dopamine agonistdose when patients develop this adverse drug reaction. Binge eating was evaluated only using The Questionnaire for impulsive-compulsive disorders in Parkinson’s Disease (QUIP)and not validated criteria, which may constitute a limitation.

In contrast with previous studies,^9, 21^ we found physical activity was directly and positively related to body weight. Yale Physical Activity Survey (YPAS)is a subjective scale measuring physical activity, validated in the general population,^22^ but not in PD. It is commonly believed that physical activity leads to increased energy expenditure, reducing body weight. Alternatively, increased activity might also reflect better health, which could in turn be related to increased body weight (also known as “the obesity paradox”).^23, 24^ YPAS has seldom been used in PD and we were unable to assess resting metabolic rate, which is a more specific marker of energy expenditure. Therefore, data from this study did not clarify the relationship between PD, physical activity, energy expenditure, and weight.

We found no correlation between body weight, nutritional status and olfaction dysfunction, in contradiction with claims by Sharma et al.^10^ who found patients with greater olfaction impairment (anosmia) had lower body weight, and patients with hyposmia were more prone to gain weight. These results are not easy to explain and have not been replicated. Our results further suggest that hyposmia does not affect body weight, while other factors commonly associated with hyposmia, such as PD severity or longer disease duration^7, 8^ were indirectly related to body weight.

We did not observe correlation between nutritional status and dysphagia, not altogether surprising, considering no late-stage PD patients were included in the study.

Antidepressant therapy has been associated with weight gain, in spite of side effects such as constipation.^25^ Our results showed negative correlation between depression and body weight, an effect dissipated in the multivariate analysis, which might be explained by the fact that study patients only presented mild to moderate signs of depression.

In conclusion, we found nutritional status, binge eating, and physical activity were the only factors directly influencing body weight. Disease duration, UPDRS II + III score and cognitive function were indirectly associated with body weight. Therefore, nutritional status should be routinely assessed in advanced PD patients with weight loss in order to recognize malnutrition and prevent its consequences. Nutritional status assessment should ideally include anthropometric measurements, nutrition screening tools, like MNA and biochemical measurements of serum protein, micronutrients, and metabolic parameters. Furthermore, it is advisable to investigate binge eating in PD patients with weight gain. Although physical activity and energy expenditure are also significant in relation to body weight regulation, further studies are needed to characterize their exact role in PD patients.

## Methods

### Study sample

One hundred fourteen consecutive unselected PD patients, diagnosed following UK Parkinson’s Disease Society Brain Bank criteria^26^ were prospectively recruited from an outpatient Movement Disorders clinic between November 2013 through September 2015. Patients with dietary restrictions, history of gastrointestinal or endocrine diseases affecting body weight, chronic infection, malignant disease, or functional brain surgery, signs of a typical parkinsonism, dementia, psychosis or who had suffered head trauma with loss of consciousness were excluded. In addition, patients referring history of drug abuse, nasal surgery, chronic sinusitis, current rhinorrhea, smoking or who had significant exposure to volatile substances, were also excluded. Patients with treated thyroid disease, balanced hormone levels or controlled diabetes mellitus were not excluded, nor were patients with gastrointestinal symptoms related to PD.

### Clinical assessment

PD patients were evaluated during the ON state, and medications with known effects on body weight recorded. Levodopa Equivalent Daily Dose (LEDD) was calculated according to conversion formulae.^27^ Clinical assessment included Movement Disorders Society-Unified Parkinson’s disease rating scale (MDS-UPDRS parts I to IV) and Hoehn & Yahr (H&Y) staging. Weight was recorded in kilograms on calibrated scales in barefoot and lightly dressed patients. Body mass index (BMI) was calculated as weight in kilograms divided by height in meters-squared. Patients were categorized according to BMI as underweight (<18.5 kg/m^2^), normal weight (18.5–24.9 kg/m^2^),overweight(25–29.9 kg/m^2^) and obese (>30 kg/m^2^).Nutritional status was evaluated using the MNA questionnaire, in which scores < 17 identify patients as malnourished, and scores between 17 and 23.5 points indicate risk of malnutrition. Energy expenditure related to physical activity was determined by using the YPAS, a questionnaire used to assess time spent conducting several activities during a typical week. Duration of each activity was multiplied by an intensity score and then added to other activities to obtain a total energy expenditure index (Kcal/day). ROME III diagnostic criteria were used to diagnose functional constipation, and dysphagia was assessed with MDS-UPDRS Part II item 3. Olfactory function was evaluated using the extended version of the Sniffin’ Sticks Test (Burghart Messtechnik, Wedel, Germany),^28^ which consists of three subtests in which felt-tip whiteboard markers are used for different olfaction modalities including threshold, discrimination, and identification. Depression was rated using the Beck Depression Inventory (cut-off score: 17). Montreal Cognitive Assessment was performed in all patients to rule out dementia (cut-off score: 24). The QUIP was used to screen for impulse control disorders, including pathological gambling, hypersexuality, excessive buying, binge eating, and compulsive medication use. Parkinson’s disease sleep scale 2 (PDSS-2)was used for sleep assessment. Study protocol conformed to Helsinki Declaration principles and was approved by the local institutional review board. All participants gave written informed consent prior to study entry.

### Statistical analysis

Descriptive data is presented as mean ± standard deviation or proportions. Alpha was set at 0.05 and Pearson’s bivariate correlations performed. Multivariate testing was conducted using SEM, which assesses the difference between an observed covariance matrix and a hypothetical predefined model covariance matrix^18, 29^ (i.e., associations between all possible pairs of variables). In other words, SEM evaluates how well a pre-specified model of postulated relationships between pairs of variables, “fits reality”. In this study, model validity was assessed by chi-square statistic, root mean square error of approximation (RMSEA), comparative fit index (CFI) and normed fit index (NFI). Models with RMSEA < 0.08, and CFI and NFI > 0.8 were considered as having good predictive power.

Results from SEM are presented in terms of age-adjusted, gender-adjusted, and height-adjusted swr between selected pairs of variables. Such coefficients represent the strength of the correlation between any pair of variables, independent from confounding factors, similar to the more commonly used partial correlation coefficient. IBM SPSS ® Statistics v.23 and AMOS ® v.23 software (Crawfordville, FL, USA) were used for analyses.
